# New Modifiable Risk Factors Influencing Coronary Artery Disease Severity

**DOI:** 10.3390/ijms25147766

**Published:** 2024-07-16

**Authors:** Kamila Florek, Maja Kübler, Magdalena Górka, Piotr Kübler

**Affiliations:** 1 Student Scientific Group of Invasive Cardiology, Institute of Heart Diseases, Wroclaw Medical University, 50-369 Wroclaw, Poland; 2 Institute of Heart Diseases, University Hospital, 50-556 Wroclaw, Poland; 3 Department of Cardiology, Faculty of Medicine, Institute of Heart Diseases, Wroclaw Medical University, 50-367 Wroclaw, Poland

**Keywords:** coronary artery disease, SYNTAX score, ischemic heart disease, atherosclerosis, inflammation, microbiota, vitamin D, obstructive sleep apnea

## Abstract

Cardiovascular diseases (CVDs) remain the leading cause of death worldwide with coronary artery disease (CAD) being the first culprit in this group. In terms of CAD, not only its presence but also its severity plays a role in the patient’s treatment and prognosis. CAD complexity can be assessed with the indicator named the SYNTAX score (SS). A higher SS is associated with major adverse cardiovascular event (MACE) occurrence in short- and long-term observations. Hence, the risk factors affecting CAD severity based on SS results may help lower the risk among patients with already developed CAD to reduce their impact on coronary atherosclerosis progression. The well-established risk factors of CAD are consistent with those associated with the coronary plaque burden. However, recently, it was shown that new indicators exist, which we present in this paper, that significantly contribute to CAD complexity such as inflammatory parameters, C-reactive protein (CRP), ratios based on blood smear results, and uric acid. Moreover, microbiota alteration, vitamin D deficiency, and obstructive sleep apnea (OSA) also predicted CAD severity. However, sometimes, certain indicators were revealed as significant only in terms of chronic coronary syndromes (CCSs) or specific acute coronary syndromes (ACSs). Importantly, there is a need to apply the interdisciplinary and translational approach to the novel CAD severity risk assessment to maximize the impact of secondary prevention among patients at risk of coronary atherosclerosis progression.

## 1. Introduction

Cardiovascular diseases (CVDs) remain the leading cause of death worldwide. In 2022, the global burden of CVDs was estimated from 73.6 per 100,000 in high-income Asia Pacific to 432.3 per 100,000 in Eastern Europe [1]. Even though CVD mortality decreased by 34.9% in the recent 30 years, ischemic heart disease (IHD) was responsible for the highest global age-standardized disability-adjusted life-years (DALYs) at 2275.9 per 100,000 [1].

IHD, also known as coronary artery disease (CAD), is commonly defined as a pathological state in which epicardial arteries are affected by atherosclerosis observed as plaque formation [2]. CAD is divided into acute coronary syndrome (ACS) and chronic coronary syndrome (CCS) presentations [2]. Whereas CCS is related to stable angina, ACS is mainly characterized by unstable angina (UA) or myocardial infarction (MI) [3]. The risk factors of CVDs were defined for the first time in the Framingham Heart Study, which is still ongoing [4]. Currently, the well-established modifiable risk factors of CAD include hypertension, hyperlipidemia, diabetes, obesity, smoking, poor diet, sedentary lifestyle, and stress [5], whereas non-modifiable risk factors are related to age, gender, ethnicity, and family history of CAD [5]. It was shown that the higher burden of coronary atherosclerosis is the most important risk factor for ACS recurrence and increases mortality after percutaneous coronary interventions (PCIs) [6,7,8,9]. A CAD complexity assessment is possible with invasive coronary angiography and computed coronary angiography [10,11]. Importantly, CAD severity is also enhanced by the presence of standard risk factors of CVDs [12]. However, there exists a residual risk of recurrent vascular events, which is present despite the optimal treatment and goal achievement in terms of cholesterol, blood pressure, and glycemia management [13,14]. Thus, there is a need to evaluate the role of new indicators suspected to impact coronary atherosclerosis complexity such as inflammation, intestinal microbiota alteration, vitamin D level, and obstructive sleep apnea (OSA) to reduce the aforementioned residual risk maximally. As not only the presence but also the severity of CAD impacts patients’ prognoses, it is crucial to focus on the risk factors affecting CAD severity, which will be helpful in secondary prevention of coronary artery plaque progression.

Hence, this review aimed to highlight new potential modifiable risk factors influencing CAD severity.

## 2. Syntax Score

The SYNTAX score (SS) is a tool that assesses multivessel CAD patients by stratifying them into low-, moderate-, and high-risk groups for major adverse cardiovascular events (MACEs) based on the anatomic complexity of plaques in the coronary arteries [10]. Higher SS values represent a bigger therapeutic challenge as well as a worse patient prognosis [10,11]. SS characterizes the extent of CAD based on a coronary angiogram assessment [10]. Firstly, the arterial dominance is determined, and then other parameters such as lesion location and diseased segments within the lesion are counted [11]. Moreover, the determination of the score involves the following classifications: chronic total occlusion, tri/bifurcation, aorto-ostial stenosis, tortuosity, presence of calcification, thrombi, diffuse disease, and lesion length > 20 mm [11]. Online tools are available for SS assessment (e.g., http://www.syntaxscore.com/ (accessed on 3 July 2024) [11]. The most commonly used cut-offs of SS for several risk groups are 0–22 for low-risk individuals, >22 for moderate-risk groups, and >33 for high-risk patients [15,16].

SS was introduced to improve the decision-making process in terms of choosing the favorable revascularization strategy between PCI and coronary artery bypass grafting (CABG) [17]. Even though the anatomic SS was related to good performance, its improvement with the involvement of clinical features such as comorbidities assessment was proposed [18]. Namely, the extended evaluation with SYNTAX Score II (SSII), apart from anatomic characteristics of CAD, includes other clinical parameters such as age, gender, left ventricle ejection fraction, creatinine clearance, presence of unprotected left main disease, chronic obstructive pulmonary disease, and peripheral vascular disease [19,20]. It was shown that SSII is associated with better risk stratification than SS in the revascularization of an unprotected left main artery [21]. However, both scores revealed a significant validation of CAD severity and MACE prediction in short-term and long-term follow-ups [8,22,23,24,25,26].

## 3. Inflammation Biomarkers

Inflammation contributes significantly to the development of atherosclerosis, and the prognostic role of several inflammatory markers in CAD severity has been studied. The role of inflammation in atherosclerosis etiology was confirmed in the genome-wide meta-analysis of 11.6 million variants by Chen et al. [27]. Elevated inflammatory markers are linked to a worse clinical outcome in CAD, as chronic inflammation plays a role in all phases of atherosclerosis [28]. Hence, inflammation plays a role in the primary and secondary prevention of CAD. However, by reducing the inflammatory response, it is possible to minimize its influence on atherosclerosis development and progression [29,30,31].

### 3.1. C-Reactive Protein (CRP)

CRP is a widely available and affordable indicator of inflammation severity [32]. Its role in atherosclerosis development is unlikely, but it was shown to be a predictor of CAD development [33]. CRP is reported to be similarly significant in the risk assessment of CAD as standard risk factors such as total cholesterol, non-HDL cholesterol, and arterial systolic blood pressure [33,34].

However, the role of CRP in terms of CAD severity is not widely studied. Among patients with ACS, it was shown that CRP predicted SS and the risk of stent thrombosis [35]. Another retrospective study confirmed the association among elevated serum Fe, CRP, and SS in an ACS group. Even though other well-studied parameters such as glucose, creatine, LDL, and triglyceride were included in the analysis, the strongest correlation for the marker of CAD severity was noted between CRP and Fe levels [36].

Importantly, CRP increased in ACS following myocardium injury and was shown to be substantially more often elevated among patients with ACS than those with stable CAD [37,38]. The study by Liu et al. based on the results of 10,020 patients showed that hs-CRP >10.00 mg/L was an independent predictor of CAD severity. However, this conclusion was based on the whole study including 55% of ACS patients and 44% of patients with stable CAD. When focusing on the subgroup analysis, the aforementioned cut-off predicted intermediate–high SS (>22) in patients with ACS in both considered subgroups—ST-segment elevation myocardial infarction (STEMI) and non-ST-segment elevation myocardial infarction (NSTEMI) patients. However, hs-CRP > 10.00 mg/L was not significant according to the patients with stable CAD [38].

Consequently, CRP is assumed to be a predictive factor of CAD burden. However, it was revealed only in the cohorts with ACS.

### 3.2. Indicators Involving Neutrophil Levels

Another indicator of systemic inflammation that is considered to be associated with CAD is a high neutrophil level [39]. Neutrophils are the most abundant group among leukocytes that take part in atherogenesis, plaque destabilization, and erosion [40].

In atherogenesis, neutrophil degranulation causes macrophage recruitment and reactive oxygen species (ROS) production, leading to endothelium dysfunction [39]. Consequently, LDL cholesterol accumulation occurs and then myeloperoxidase (MPO) secreted by neutrophils causes LDL oxidation with subsequent foam cell formation [39]. Increased bone marrow activation and inflammation can be predicted by peripheral blood immature neutrophil/granulocyte (IG) levels. It was shown that IG is significantly increased among patients with higher SS. Moreover, a cut-off value of 0.7 was shown to represent 76.2% sensitivity and 75.4% specificity in terms of high SS prediction [41].

An interesting indicator that is considered useful in terms of risk assessment in patients with CAD is the neutrophil–lymphocyte ratio (NLR) [42]. Importantly, lymphocytes play a regulatory role in the atherogenesis process [43].

However, NLR’s role in CAD severity is still under debate. Li et al. published a meta-analysis showing that NLR was significantly associated with the complexity of CAD for the cut-off ranging from 1.95 to 3.97 [44]. However, some newer studies confirmed that NLR is related to CAD severity in ACS patients, where they indicated more potent risk factors [45,46]. Even though NLR was associated with SS, in the Maleki et al. study, the TIMI risk score was found to be a better predictor of atherosclerosis burden [45]. Another study by Mohanty et al. investigated the role of NLR, monocyte-to-high-density lipoprotein cholesterol ratio (MHR), and monocyte–lymphocyte ratio (MLR) in CAD severity prediction and showed MHR superiority among the other considered factors [46]. Furthermore, NLR was shown to predict residual SS among patients after STEMI [47]. In addition, where CAD and cancer were observed to co-exist in the same individuals, it was shown that cancer was associated with higher SS, whereas this association was stronger among patients with cancer and higher inflammation described by NLR [48,49].

However, one study questioned the role of NLR in CAD severity prediction in stable CAD by revealing the significant role of the lymphocyte-to-monocyte ratio (LMR) [50]. Even though NLR was rejected as an SS predictor in the Rostami et al. study, it was shown to be useful in the no/slow flow phenomenon risk assessment in STEMI patients [51]. Importantly, another study rejected NLR’s role as a predictor of CAD complexity in the general population, where NLR was found to be an independent predictor of SS only in males [52].

Therefore, a comparison of NLR’s role with other available indicators based on blood count results should be performed in meta-analyses to obtain an optimal marker for CAD severity prediction, which would be helpful in risk stratification.

### 3.3. Systemic Immune-Inflammation Index (SII)

Another recent indicator is SII, calculated as (neutrophil × platelet)/lymphocyte levels [53]. Neutrophil and lymphocyte roles were predefined, whereas platelets’ role in atherosclerosis relies on sticking to the vessel wall, promoting leukocyte aggregation, and starting the atherosclerotic process before leukocytes enter the atherosclerotic plaque [54].

SII was shown to be a potential indicator of cardiovascular disease and is associated with higher mortality among patients with CAD [55,56]. Furthermore, the association between SII and the burden of CAD was also recently studied. In the study by Candemir et al., SII was shown to be significantly associated with high SS among patients with stable angina pectoris. Moreover, the cutoff value of 750 × 10^3^ predicted severe coronary lesions with a sensitivity of 86.2% and specificity of 87.3% [57]. Therefore, in ACS patients, it was shown to predict CAD severity as well as acute stent thrombosis [52,58]. However, in another study, a novel systemic immune-inflammation response index (SIIRI) was examined, calculated as peripheral neutrophil × monocyte × platelet ÷ lymphocyte count [59]. The optimal cut-off for SIIRI was 4.3 × 10^5^, with sensitivity = 69.9% and specificity = 75.8%. However, in that study, SIIRI had a higher area under the ROC (receiver operator curve) as compared with SII, NLR, MLR, and PLR (platelet–lymphocyte ratio) [59].

### 3.4. Uric Acid (UA)

Hyperuricemia has been proven to predispose to atherosclerosis [60]. The high intracellular concentration of UA enhances the expression of inflammatory markers, such as nuclear factor κB (NF-κB), growth factors, and vasoconstrictive substances, and induces the proinflammatory response of macrophages [61]. Moreover, UA stimulates the proliferation of smooth muscle cells, promotes endothelium dysfunction, and increases oxygen-free radical production, causing low-density lipoprotein oxidation [61,62,63]. On the other hand, lower serum albumin synthesis and elevation in its catabolism have been related to an elevated inflammatory response [64].

While the correlation between hyperuricemia and increased severity of CAD was previously revealed, a novel indicator was investigated—the uric acid-to-albumin ratio (UAR) [65]. Among NSTEMI patients, UAR was shown to predict SS for intermediate–high severity groups better than NLR, which was found to be insignificant in the analysis [66]. However, when the study population included STEMI, NSTEMI, and unstable angina (UA) patients, the predictive value of UAR was not visible [66].

As the aforementioned indicators are widely available in reasonable conditions, they should be considered potential biomarkers of the higher risk of severe CAD. Sometimes, these biomarkers are observed to affect the ongoing inflammatory process; thus, the underlying causes should be identified and treated.

### 3.5. Anti-Inflammatory Therapy as a Secondary Prevention of CAD

Some data claim that anti-inflammatory therapy can significantly reduce the rate of recurrent cardiovascular events [29,30,31]. This was proven in randomized clinical trials according to the influence of colchicine and canakinumab on cardiovascular risk [29,30,31]. Canakinumab selectively inhibits interleukin-1β, while colchicine has a broad mechanism of action affecting tubulin polymerization, microtubule generation, and leukocyte responsiveness [30,31]. In the CANTOS trial, it was shown that canakinumab at a dose of 150 mg administered subcutaneously every three months significantly reduced the risk of recurrent cardiovascular events independently of lipid management [29]. Furthermore, in the COLCOT trial, patients who received 0.5 mg of colchicine daily were at a lower risk of recurrent ischemic events after a prior MI than those in the placebo group [31]. Moreover, in the LoDoCo2 among patients with chronic coronary disease, the same scheme of colchicine administration was shown to reduce the risk of cardiovascular events [30]. Based on the COLCOT and LoDoCo2 trials, European Society of Cardiology guidelines considered low-dose colchicine for the secondary prevention of CAD among high-risk patients (class IIb recommendation—level of evidence A) [67]. Hence, these encouraging results confirm that the role of anti-inflammatory drugs in CAD secondary prevention should be studied in further research. 

## 4. Microbiota Alternation

Chronic inflammation may also be associated with underlying causes that are not widely known. Recently, a substantial peak in data about the role of the gut microbiome in cardiovascular diseases was observed [68]. Hence, an “imbalance” in the gut microbiota is commonly defined as dysbiosis, which can be driven by changes in quality or quantity in the bacteria abundance in the gut [69,70]. The potential mechanism underlying dysbiosis involvement in cardiovascular pathologies is low-grade inflammation, which may significantly contribute to atherosclerosis formation [71,72]. Moreover, the data highlight the role of microbiota composition and its metabolites in heart–gut crosstalk [73]. Interestingly, it was reported that the microbial community not only differs between patients with CAD and without its signs, but it can also distinguish between patients with stable angina and those with ACS [69,74]. In terms of microbial metabolites, the meta-analysis by Ottosson et al. revealed that among 33 different microbial metabolites characterized using 16S rRNA sequencing, only phenylacetylglutamine (PAG) was a predictor of CAD risk independent from other cardiovascular risk factors [75]. However, understanding the impact of gut microbiota variations on CAD is currently mainly limited to small cohort analyses, showing concise data about taxa-specific roles in CAD severity [76,77]. The impact of fungal communities on CAD was also revealed; however, it seems to be underrated in the current studies [78].

### 4.1. Lactobacillus

The most convincing data about specific genera influencing atherosclerosis was found from *Lactobacillus,* which was detected to influence CAD severity [79]. The potential mechanism underlying that effect may be associated with *Lactobacillus gasseri*, whose supplementation lowered triglyceride and LDL-cholesterol levels playing a crucial role in atherogenesis [80]. Importantly, higher *Lactobacillus* abundance lowered the risk of MACEs and all-cause death [79]. In a case-control study, *Lactobacillus* was detected by 16S rDNA sequence analysis, and its levels were determined by real-time PCR among 402 ACS patients and 100 controls. In the subgroup analysis, higher *Lactobacillus* levels were significantly associated with a lower risk of SS > 33 among STEMI patients, whereas in NSTEMI and UA patients, these results were not noted [79]. However, another study that also used 16S rDNA sequence analysis to detect *Lactobacillus* among 360 ACS patients found an association between higher coronary atherosclerosis burden and lower *Lactobacillus* abundance among the whole cohort of ACS patients [81]. Moreover, the aforementioned study also confirmed the role of lower total bile acid (TBA) serum levels on CAD severity and showed it is common with a negative predictive role of Lactobacillus in all-cause death and cardiac death [81].

### 4.2. Trimethylamine N-Oxide (TMAO)

Mounting evidence suggests the impact of TMAO on atherosclerosis development. TMAO is a plasma metabolite produced by the human microbiome. First, trimethylamine (TMA) from choline, phosphatidylcholine, and l-carnitine is formed, then, through the portal circulation, TMA is delivered to the liver, where it is converted into TMAO [82]. The potential mechanism by which TMAO exacerbates calcified plaque formation may be driven by enhancing the inflammation of the vascular wall, inducing ROS production, inhibiting NO synthesis, and reversing cholesterol transport [83]. A meta-analysis by Yao et al. found that elevated TMAO plasma concentrations increased the risk of MACEs among patients with CAD, with a cut-off of 5.1 μmol/L for this prediction [84]. In terms of coronary artery lesion complexity, TMAO was not independently correlated with the presence of CAD or the severity of coronary atherosclerosis in the included population. Nevertheless, a significant association between circulating TMAO and higher coronary atherosclerotic burden was observed in patients with eGFR lower than 60 mL/min/1.73 m^2^ [85]. Another study confirmed that TMAO was an independent predictor of multivessel disease and intermediate–high-risk SYNTAX after adjustment for traditional risk factors [86]. Furthermore, a prospective SZ-NSTEMI trial may elucidate the relationship between TMAO and the severity and prognosis of coronary atherosclerosis in newly diagnosed NSTEMI patients who will undergo SS assessment. Moreover, a follow-up within 1 month, or 12 months according to MACE rates, will be performed [87]. Additionally, among patients with stable angina, fasting plasma TMAO also correlated positively with coronary artery disease complexity [88]. Of note, TMAO levels change in the general population and were shown to be significantly higher among men [89]. In the patients with cerebral ischemic events with the underlying atherosclerotic mechanism, those who were older males with lower eGFR had higher TMAO and TMAO levels that did not differ significantly according to different races and ethnic groups [89].

Lowering TMAO levels is possible by reducing the intake of its precursor, TMA, in the diet with the exclusion of red meat [90]. Moreover, in randomized controlled trials (RCTs), a cohort study showed that pharmacological interventions with rifaximin were effective in TMAO reduction as well as with statin use [91,92].

### 4.3. Phenylacetylglutamine (PAG)

**Figure 1:**
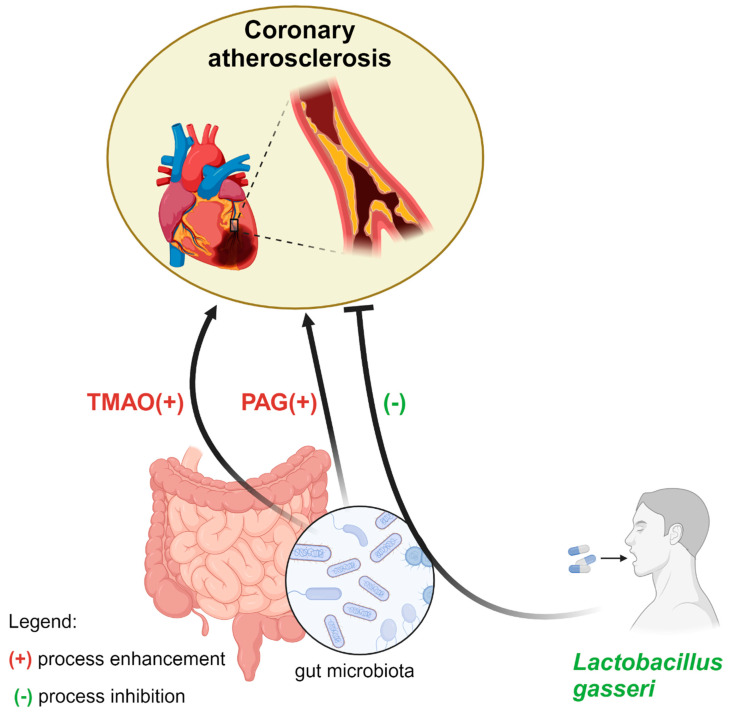
Coronary atherosclerosis burden associations with *Lactobacillus gasseri* supplementation, TMAO, and PAG. (+)—enhancement, (-)—inhibition.

Another gut microbiota metabolite—the aforementioned PAG—was found in the Ottoson et al. meta-analysis to increase the risk of future CAD independently of other cardiovascular risk factors [75]. PAG was elevated among patients with a high atherosclerotic plaque burden, which was unexplainable with its traditional risk factors [93]. However, PAG involvement in atherosclerosis has not been elucidated yet. Some studies suggest that an increase in PAG may be associated with age, hypertension or diabetes, and kidney impairment; thus, in that scenario, it would just reflect the presence of standard risk factors [94]. Nonetheless, PAG has recently been found to be associated with MACEs. Moreover, an independent association was found between plasma PAG levels and the coronary atherosclerotic burden among patients with suspected CAD [93]. Currently, PAG may be considered a potential novel biomarker of CAD burden. However, there is no therapeutic strategy to lower PAG, except the treatment of the underlying disease; thus, it should be evaluated in further research (Figure 1).

Additionally, a currently ongoing prospective CorLipid Trial is trying to evaluate the role of several biomarkers in CAD severity prediction. These involve ceramides, acyl-carnitines, fatty acids, and proteins such as galectin-3, adiponectin, and the ratio of apolipoprotein B/apolipoprotein A1 [95]. Of note, the majority are involved in the microbiota metabolic pathways [96,97,98].

Overall, further research on the gut–heart crosstalk is needed to elucidate the harmful and protective impact of several taxa and metabolites on CAD development and burden.

## 5. Vitamin D

Another new risk factor that is revealed to be associated with CAD is vitamin D. Vitamin D belongs to the group of fat-soluble vitamins. Its production in the body is initiated by exposure to ultraviolet B rays. Additionally, it can be obtained from a proper diet, mainly from animal-derived products. Vitamin D acts as a prohormone and undergoes a series of metabolic transformations in the body, leading to the formation of its biologically active form—1,25-dihyroxyvitamin D. It functions by binding to a group of nuclear receptors called vitamin D receptors [99].

The role of vitamin D deficiency in the pathogenesis of cardiovascular diseases, including CAD, appears to be important and probable. The mechanism of action is based on a series of processes and their modulation. Receptors for 1,25-dihydroxyvitamin D are found in smooth muscle cells, suggesting its influence on their contraction and thus blood pressure regulation. Vitamin D likely primarily prevents hypertension through three mechanisms—by directly suppressing the renin–angiotensin system, inhibiting extracellular matrix accumulation in vessel walls, and reducing arterial stiffness by enhancing nitric oxide (NO) synthesis by the endothelium. Additionally, vitamin D inhibits cholesterol uptake by macrophages, preventing foam cell formation and their accumulation in vessel walls, thus preventing atherosclerosis development [100].

It has also been demonstrated that vitamin D deficiency promotes the secretion of pro-inflammatory cytokines such as IL-6 and TNF-alpha, which play a crucial role in plaque rupture. Pathologies such as insulin resistance and pancreatic β-cell dysfunction, classified as independent risk factors for CAD, have also been associated with vitamin D deficiency [101]. Although the association between vitamin D deficiency and the tendency to develop cardiovascular diseases appears to be significant, there is a lack of clear data regarding the direct correlation between vitamin D levels in the body and the development of CAD.

In one study, the relationship between vitamin D levels and risk factors of CAD was examined. Patients with cardiac symptoms were divided into three groups based on their vitamin D levels. In the group with the lowest vitamin D level (<20 ng/mL), a significantly higher prevalence of diabetes and triple-vessel disease was observed. Moreover, a significant negative correlation was demonstrated between vitamin D and HbA1c levels—HbA1c is considered an independent risk factor for CAD. However, no significant correlation was found between the vitamin D level and higher SS [101]. Another study retrospectively reviewed the medical records of patients who underwent coronary artery bypass graft surgery; serum vitamin D level was not found to be an independent predictor of higher SS [102]. On the other hand, some studies show a significant negative correlation between 25(OH)D levels and SS and identified 25(OH)D as an independent factor related to higher SS and severity of coronary artery stenosis [103]. It has also been indicated that in patients with STEMI/NSTEMI, serum 25(OH)D levels were significantly lower, which might cause the absence of spontaneous reperfusion that leads to increased disease severity.

**Figure 2:**
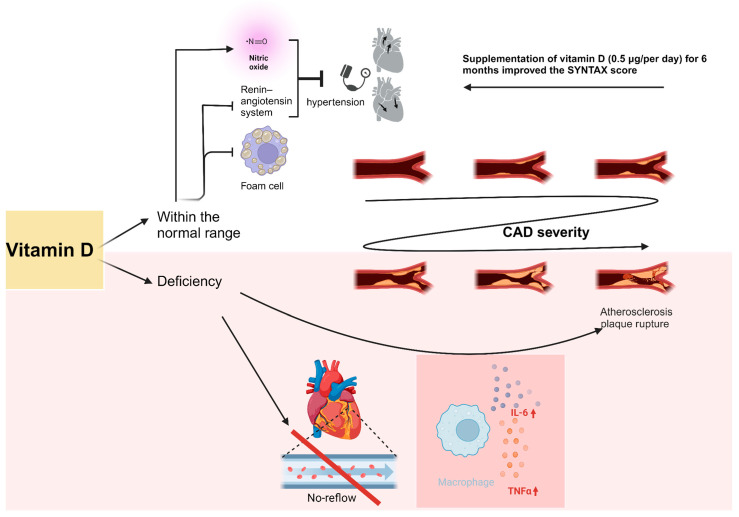
The impact of vitamin D on CAD severity.

Moreover, vitamin D deficiency was found to be an independent predictor of the no-reflow phenomenon after percutaneous coronary intervention in patients with STEMI/NSTEMI [104,105]. Vitamin D levels may also correlate with CAD extent and complexity assessed by SS [106]. In another study, patients with CAD were asked to supplement vitamin D (0.5 μg/per day) for 6 months. After 6 months of treatment, an improved SS was noticed, which led to the conclusion that vitamin D supplementation might be a beneficial factor in the secondary prevention of CAD (Figure 2) [107].

Vitamin D within the normal range, defined as 30–50 ng/mL, inhibits the renin–angiotensin system and promotes NO synthesis, which has an antihypertensive effect and thus reduces the occurrence of CAD standard risk factor [100]. Normal ranges of vitamin D inhibit foam cell formation, which reflects its antiatherogenic effect [100]. One study showed a reduction in coronary atherosclerosis burden after 6 months of supplementation; however, these results should be confirmed in further studies [107]. Vitamin D deficiency was shown to increase the risk of the no-reflow phenomenon after PCI [104,105]. Moreover, via increased TNF-alpha and il-6 production, vitamin D level deficiency promotes atherosclerotic plaque rupture [100].

Based on the risk of CAD development, not only its severity, the meta-analysis by Tabaei et al. and Alizadeh et al. revealed that vitamin D receptor (VDR) gene polymorphism plays a role in CAD susceptibility [108,109]. This association varies among populations with the significant involvement of FokI and ApaI SNP polymorphisms in the European and Asian populations [108]. However, there is a lack of VDR gene polymorphism analysis according to CAD severity.

In summary, the relationship between vitamin D levels and CAD severity is a complex and equivocal issue. Although some studies show a significant correlation between these factors, many of them exclude a direct influence. Further research on this topic is still required. However, vitamin D deficiency increases the risk of cancers, autoimmune diseases, hypertension, and infectious diseases [110]. Thus, independently from vitamin D’s neutral or negative effect on CAD progression, patients should have vitamin D within the normal range as its deficiency is associated with a variety of complications.

## 6. Sleep Apnea

Another interesting indicator related to CAD development and severity is OSA—a disorder in which a collapse of the upper respiratory tract leads to respiratory arrest and sleep interruption [111]. Studies conducted in recent years have shown that there is a connection between OSA and CAD; however, there is controversy in establishing this relation [112,113]. It was revealed that patients suffering from mild OSA were characterized by a significantly lower SS compared with those who were affected by moderate-to-severe OSA [112].

Another study investigated the influence of OSA on CAD exacerbation in the Chinese population. Patients suffering from OSA were characterized by an apnoea–hypopnoea index (AHI) of at least 5/h. The investigation revealed a significantly more frequent presence of OSA in CAD patients. Moreover, the study exposed a higher severity of CAD in patients with OSA in comparison with those who did not struggle with such respiratory problems. The results of the study showed that OSA alone is an important risk factor in the analyzed population and should be taken into consideration during the prevention and treatment of CAD [114].

Even though many studies prove the connection between OSA and CAD severity, others contradict this concept [115]. The meta-analysis by Xie et al. evaluated studies including patients with sleep apnea–hypopnea syndrome in terms of its correlation with cardiovascular risk [116]. It was shown that sleep apnea was associated with more complex coronary artery lesions [116]. Moreover, it was shown that continuous positive airway pressure (CPAP), which is used in OSA treatment, reduced the cardiovascular risk according to repeat revascularization, myocardial infarction, stroke, and cardiovascular mortality [117].

The suspected mechanisms of OSA’s impact on CAD development are complex and include oxidative stress, endothelial dysfunction, and inflammatory and immunologic factors [118]. Repeated hypoxia and reoxygenation among OSA patients lead to endothelial dysfunction by increasing oxidative stress, which can be reduced with CPAP therapy [119]. Moreover, oxidative stress is associated with lipid peroxidation, thus enhancing atherosclerosis development [120].

The relationship between OSA and CAD becomes more complicated when we take the risk factors of OSA into consideration, as these risk factors can be seen as confounders. The most important risk factor of OSA is obesity, which plays a role in CAD development as well; however, in OSA, the correlation with obesity is linear [121]. The influence of obesity in OSA includes changes in the anatomy of the upper respiratory tract, which is narrowed by fat deposits [122]. Moreover, the metabolic activity of fat tissue leads to the release of inflammatory cytokines, which also may be related to the pathogenesis of OSA [123].

Obesity not only contributes to the occurrence of OSA but also to the CAD manifestation. The pathogenesis is similar to the one in OSA—adipose tissue, as a metabolically active area, releases many proinflammatory cytokines, such as leptin, resistin, IL-6, and monocyte chemoattractant protein, which may participate in the process of coronary artery wall hardening and contribute to the development of CAD [122,123]. Moreover, the risk of OSA is also higher among patients with the other traditional risk factors of CAD—male sex or smoking [124].

Additionally, hypertension is a standard risk factor for CAD, whereas it is highly associated with OSA. Even after controlling for potential confounders—such as age and obesity—OSA coexists in 50% of patients with hypertension [125].

However, according to CAD development, there is not enough data from the group of non-obese patients with OSA (NOOSA), which would be beneficial in minimizing the confounding effect of obesity. It was shown in the cohort study by Luyster et al. that the risk of coronary artery calcifications was significantly increased among NOOSA patients [126]. Further research involving NOOSA patients is required to elucidate if there is a direct correlation between OSA and CAD.

Nevertheless, not all risk factors of OSA should be considered equivalent to risk factors of CAD; an example is alcohol consumption. A significant relationship was found between alcohol intake and risk as well as the severity of OSA. This relationship was notable, especially among women, who were characterized by a significant association between alcohol consumption and AHI [127]. However, such a connection was not observed in CAD. A study among Million Veteran Program participants revealed a reduced risk of CAD in those who consumed alcohol lightly to moderately [128].

Moreover, for a consistent ethanol intake of up to 48 g, the frequency of alcohol consumption per week showed a negative correlation with CAD risk [129]. However, it is necessary to mention that heavy alcohol drinking episodes increase the risk of MI [130]. The above example of alcohol consumption is important in the context of determining the risk factors of CAD and OSA. Even though many of the risk factors of OSA and CAD are common, some of them are contrary, and more studies involving patients without confounding factors may be helpful in clarifying this association. 

Overall, the impact of OSA on CAD severity should be further evaluated and established. However, a therapeutic option for that disease—CPAP—gives promising insight into the modification of this potential primary and secondary risk factor (Table 1).

## 7. Conclusions

As CAD severity influences the patient’s prognosis, potential novel risk factors of the coronary atherosclerosis burden should be further evaluated. Some of them can be modified and considered in the secondary prevention of CAD—such as vitamin D deficiency and OSA—and in terms of microbiota alterations, some probiotic interventions may be helpful. However, further research is required to consider some of these interventions as recommended. Interventions that can be currently implemented in practice are anti-inflammatory therapy applied consistently with the European Society of Cardiology guidelines and vitamin D deficiency, as it requires supplementation independently from its protective role in CAD progression, which is yet to be established. Moreover, as inflammatory biomarkers reflect potential risk factors affecting CAD severity, after identifying and treating the underlying cause of inflammation, its reduction may be possible. As traditional risk factors play a crucial role in the primary and secondary prevention of CAD development, the potential benefits of novel risk factor establishment and management can be mainly observed among patients at high risk of cardiovascular events despite optimal medical treatment. In terms of new risk factors of CAD, interdisciplinary and translational cardiology approaches in the prevention of CAD development and prognosis are warranted.

## Figures and Tables

**Table 1:** Summary of the potential novel modifiable risk factors of CAD severity with modification strategies.

Risk Factors		Clinical Condition of Risk Factor Significance	Modification	Study Type
Inflammation	CRP [26,27,28,29,30,31,32]	ACS	Canakinumab [29], colchicine [30,31]	RCTs-CANTOS [29], COLCOT [30], LoDoCo2 [31]
	NLR	ACS [39,40]/ declined [44]/ only in males [46]		
	SII	Stable angina and ACS [46,52]		
	UAR	NSTEMI		
Microbiota	*Lactobacillus* abundance	ACS [69,72]	*Lactobacillus gasseri* supplementation [73]	RCT [73],
	TMAO	NSTEMI [78], stable angina [79]	Red meat exclusion [90], statins [91], rifaximin [92]	RCT [90], cohort study [91], RCT [92]
Vitamin D		Stable angina [95]	Vitamin D supplementation (0.5 μg/per day) [95]	RCT [95]
Sleep apnea	OSA	CCS	CPAP [102]	RCT [102]

## Data Availability

Data sharing is not applicable as no datasets were generated or analyzed during the current study.
